# The crystal structure of mouse IRG1 suggests that cis-aconitate decarboxylase has an open and closed conformation

**DOI:** 10.1371/journal.pone.0242383

**Published:** 2020-12-01

**Authors:** Hye Lin Chun, So Yeon Lee, Ki-Hwa Kim, Chang Sup Lee, Tae-Jin Oh, Hyun Ho Park

**Affiliations:** 1 College of Pharmacy, Chung-Ang University, Seoul, Republic of Korea; 2 Department of Life Science and Biochemical Engineering, Graduate School, SunMoon University, Asan, Republic of Korea; 3 College of Pharmacy and Research Institute of Pharmaceutical Science, Gyeongsang National University, Jinju, Republic of Korea; 4 Genome-based BioIT Convergence Institute, Asan, Republic of Korea; 5 Department of Pharmaceutical Engineering and Biotechnology, SunMoon University, Asan, Republic of Korea; University of Canterbury, NEW ZEALAND

## Abstract

Itaconate, produced as an offshoot of the TCA cycle, is a multifunctional immunometabolite possessing antibacterial, antiviral, immune regulation, and tumor progression activities. The production of itaconate in biological systems is catalyzed by cis-aconitate decarboxylase (CAD, also known as immune responsive gene 1 (IRG1) in mammals). In this study, we solved the structure of IRG1 from Mus musculus (mouse IRG1). Structural comparison analysis revealed that IRG1 can exist in either an open or closed conformation and that this is controlled by the A1 loop located proximal to the active site. Our closed form structure was maintained by an unidentified molecule in the active site, which might mimic its substrate.

**Protein Data Bank accession codes:**

Coordinate and structural factors were deposited with the Protein Data Bank under PDB ID: 7BR9.

## Introduction

Itaconate, the decarboxylated product of cis-aconitate (an intermediate metabolite in the TCA cycle), is a small immunometabolite that can control innate immunity and possesses antibacterial and antiviral activity [1–3]. This small metabolic intermediate has become an increasingly prominent polymer in the industry and is used for generating various resins and bioactive compounds [4, 5]. Recent studies have highlighted the various biological functions of itaconate, focusing on immune cells [2, 6, 7]. Itaconate produced by macrophages during Salmonella enterica and Mycobacterium tuberculosis infection kills these bacteria by directly interacting with bacterial isocitrate lyase (ICL), which is a key enzyme of the glyoxylate shunt [1, 8–10]. Zika virus-infected neurons produce itaconate, which can inhibit succinate dehydrogenase (SDH) activity and viral replication, indicating that itaconate has antiviral activity [2]. SDH inhibition by itaconate also regulates the innate immunity by metabolic remodeling of immune cells [11].

**Fig 1:**
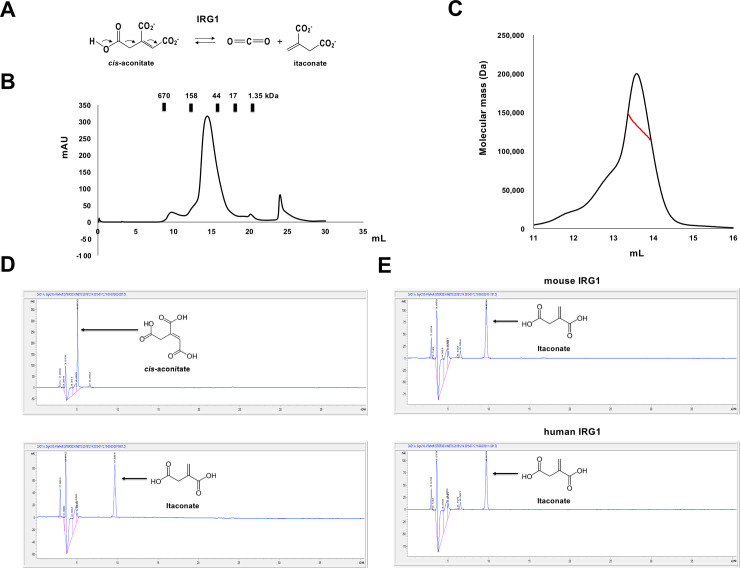
Mouse IRG1 purification and activity analysis. (A) Itaconate production catalyzed by IRG1 using cis-aconitate as the substrate. (B) Size-exclusion chromatography profile. The calibrated positions of the size markers are indicated above the profile. (C) MALS profile; the red line indicates the experimental mass size. (D) HPLC profiles. The eluted positions of cis-aconitate (upper panel) and itaconate (lower panel) are indicated by black arrows. (E) HPLC profiles showing IRG1 activity. The peak produced by the enzymatic reaction is indicated by a black arrow.

Initial studies examining itaconate production and function in the fungus Aspergillus terreus, the organism with the highest itaconate production level, have indicated that decarboxylation of cis-aconitate followed by itaconate generation is catalyzed by a ~55 kDa protein named cis-aconitate decarboxylase (CAD) [12, 13]. In mammalian systems, itaconate is most highly produced in macrophages in responce to pathogen-associated molecular patterns (PAMPs) such as lipopolysaccharide (LPS) [1, 14–16]. In mammalian systems, itaconate production is catalyzed by immune responsive gene 1 (IRG1; Fig 1A), which is known as an LPS-inducible protein highly expressed during the inflammation phase of pathogen infection [1]. IRG1 is produced by mouse Acod1 gene. As excessive, IRG1 overproduction-mediated itaconate production is linked to many diseases, including gout, chronic arthritis, and cancer, in mouse models, IRG1 has been suggested as an optimal target for therapeutic intervention [17–19].

Despite the critical roles of itaconate in the polymer industry, biomedical applications, the catalytic mechanism of IRG1 in innate immunity have remained elusive owing to the limited number of structural studies. Interestingly, at the time of preparing this manuscript, after solving and analyzing the structure of mouse IRG1, the structures of another mouse IRG1 (PDB ID: 6R6T) and human IRG1 (PDB ID: 6R6U) were reported by Chen et al. [20]. This study provides the structural information of full-length mouse IRG1 and compares this structure with that of another, recently reported mouse IRG1 (6R6T) [20]. This structural comparison revealed that the IRG1 can exist in either an open or closed conformation, which is controlled by the A1 loop located proximal to the active site. Furthermore, structural analysis revealed that maintenance of our IRG1 closed form structure was accomplished by an unidentified molecule in the active site, which might mimic its substrate.

## Materials and methods

### Protein expression and purification

The full-length Mus musculus (NCBI reference sequence ID: NP_032418.1) was synthesized by BIONICS (Seoul, Republic of Korea) and cloned into a pET21a expression vector (Invitrogen, California, USA) using a NdeI and XhoI restriction sites. The plasmid encoding the full-length mouse IRG1 was transformed into Escherichia coli BL21 (DE3) cells. A single colony was selected and cultured in lysogeny broth (LB) medium containing 50 μg/mL kanamycin overnight at 37°C. The cells were then transferred and cultured in 3 L of medium until reaching an optical density value at 600 nm of approximately 0.75, at which point 0.5 mM isopropyl β-D-1-thiogalactopyranoside was added to the medium and the cells were further cultured for 18 h at 20°C. The cells were then harvested by centrifugation at 20°C and the collected cells were washed with 40 mL of lysis buffer (20 mM Tris-HCl [pH 8.0], 500 mM NaCl, and 25 mM imidazole). After adding a serine protease inhibitor (phenylmethanesulfonyl fluoride; Sigma-Aldrich, St. Louis, MO, USA), the cells were disrupted by sonication on ice with six bursts of 30 sec and a 90 sec interval between each burst. The lysed cell suspension was centrifuged at 10,000 g for 30 min at 4°C to remove the cell debris and the supernatant was mixed with nickel nitrilotriacetic acid (Ni-NTA) resin (Qiagen, Hilden, Germany) by gentle agitation for 1 h at 4°C. The resulting mixture was applied to a gravity-flow column pre-equilibrated with lysis buffer. The column was washed with 50 mL of washing buffer (20 mM Tris-HCl [pH 8.0], 500 mM NaCl, and 60 mM imidazole). Next, a total of 2 mL of elution buffer (20 mM Tris-HCl [pH 7.9], 500 mM NaCl, and 250 mM imidazole) was loaded onto the column to elute the bound protein. The resulting eluate was concentrated to 20 mg/mL and sequentially subjected to size exclusion chromatography (SEC). SEC purification was conducted using an ÄKTA explorer system (GE Healthcare, Chicago, IL, USA) equipped with a Superdex 200 Increase 10/300 GL 24 mL column (GE Healthcare) pre-equilibrated with SEC buffer (20 mM Tris-HCl [pH 8.0] and 150 mM NaCl). Protein fractions were collected, concentrated to 8.2 mg/mL, flash-frozen in liquid N2, and stored at -80°C until use. Human IRG1, which was produced and used for the activity study, was prepared using the same method used for mouse IRG1.

### SEC-MALS analysis

The absolute molar mass of the full-length mouse IRG1 was determined by multi angle light scattering (MALS) analysis. The target protein filtered with a 0.2 μm syringe-filter was loaded onto a Superdex 200 10/300 gel-filtration column (GE Healthcare) pre-equilibrated in a 20 mM Tris-HCl [pH 8.0] and 150 mM NaCl buffer. The mobile phase buffer flowed at a rate of 0.4 mL/min at 25°C. A DAWN-treos MALS detector (Wyatt Technology, Santa Barbara, CA, USA) was interconnected with the ÄKTA explorer system (GE Healthcare). The molecular mass of bovine serum albumin was used as the reference value. Data for the absolute molecular mass was assessed using the ASTRA program (Wyatt Technology).

### Decarboxylation activity test and HPLC analysis

The cis-aconitate used as a substrate is converted to the product itaconate by IRG enzymes. The cis-aconitate was prepared by dissolving in water. For the enzymatic tests, the final 100 μL was incubated in 25 μM HEPES buffer [pH 7.1] supplied with 1.5 μM of each enzyme and 1.7 mM cis-aconitate. The reaction proceeded for 1 h at 30°C, and after the reaction it was extracted by adding 400 μL methanol [21]. The reaction mixture was analyzed using Agilent High-Pressure Liquid Chromatography (HPLC) equipped with an ACC-3000 autosampler, Mightysil RP-18 GP reverse-phase C18 column (150 V, 4.6 mm, Japan) and DAD-3000 diode array detector. The mobile phase consisted of water mixed with 0.1% trifluoroacetic acid as a solution A and HPLC-grade acetonitrile as a solution B. Substrate and their product were detected by UV detector at 210 nm, and temperature was kept at 30°C [22]. The flow rate for separating the sample was set to 0.9 mL/min, and the solution A was maintained at 95% for 40 min for analysis.

### Crystallization and data collection

For initial crystallization, 1 μL of protein solution was mixed with an equal volume of reservoir solution and the droplet was allowed to equilibrate against 300 μL of the mother liquor using the hanging drop vapor diffusion method at 20°C. The initial crystal was observed in a 20% PEG 4000, 0.1 M Tris-HCl [pH 8.5], and 0.2 M lithium sulfate monohydrate buffer. Crystallization conditions were further optimized and finally adjusted to a buffer composition of 26.5% PEG 4000, 0.085 M Tris-HCl [pH 8.2], 0.17 M lithium sulfate monohydrate, and 20% (v/v) glycerol. Qualified needle shaped crystals appeared after 9 days and grew to a maximum size of 0.5 × 0.05 × 0.05 mm^3^. For data collection, the crystals were soaked in the mother liquor supplemented with 30% (v/v) glycerol as a cryoprotectant solution and flash-cooled in a N2 stream at -178°C. X-ray diffraction data were collected at the Pohang Accelerator Laboratory with the 5C beamline (Pohang, Republic of Korea). The diffraction data were indexed, integrated, and scaled using the HKL-2000 program [23].

### Structure determination and refinement

The structure was determined using the molecular replacement (MR) phasing method with PHASER [24]. The previously solved structural homologue iminodisuccinate (IDS) epimerase (PDB ID: 2HP0), which shares 25% sequence identity with mouse IRG1, was used as a search model [25]. The initial model was built automatically with AutoBuild in PHENIX [26] and completed with Coot [27]. Model refinement was iteratively performed using phenix.refine in PHENIX [26]. Model quality was validated using MolProbity [28]. All the structural figures in this paper were generated using the PyMOL program [29].

### Protein Data Bank accession codes

Coordinate and structural factors were deposited with the Protein Data Bank under PDB ID: 7BR9.

## Results

### Overall structure of mouse IRG1

To elucidate the cis-aconitate to itaconate catalyzing mechanism of IRG1, the full-length DNA coding sequence of mouse IRG1 (NCBI reference sequence ID: NP_032418.1) was synthesized and cloned into a pET21 vector for expression in bacteria. The target protein was purified by two rapid chromatography steps using affinity chromatography followed by size-exclusion chromatography (SEC). This process produced soluble dimeric mouse IRG1, eluted at approximately 14–15 mL of the SEC profile (Fig 1B). Owing to the lack of data regarding the working stoichiometry of the IRG family, the absolute molecular mass of mouse IRG1 in solution was analyzed by MALS. The experimental molecular mass of mouse IRG1 measured by MALS was 130.2 kDa (0.42% fitting error). As the theoretically calculated molecular weight of monomeric full-length mouse IRG1 (from residues 1 to 488) including the C-terminal His-tag was 59.2 kDa, mouse IRG1 appeared to exist as a dimer in solution (Fig 1C).

Prior to crystallization for the structural study of mouse IRG1, the activity of both mouse IRG1 and human IRG1 was tested using high-pressure liquid chromatography (HPLC) to determine whether mammalian IRG1s expressed in bacteria exhibit proper activity. After determining the eluted positions of cis-aconitate and itaconate by HPLC (Fig 1D), the reaction mixture, including cis-aconitate plus mouse IRG1 or human IRG1, was injected into the HPLC apparatus and itaconate production was monitored by analyzing the eluted position of the newly produced compound. The HPLC profile showed that similar amounts of itaconate were produced by both mouse IRG1 and human IRG1, indicating that both mammalian IRG1s have cis-aconitate decarboxylase activity (Fig 1E).

Following crystallization and X-ray diffraction data analysis, we solved the structure of mouse IRG1 using the MR method. We used the structure of IDS epimerase (PDB ID: 2HP0), a previously reported structural homolog sharing 25% sequence identity with mouse IRG1 [25], for the MR search model, as it was the only structure with high structural similarity. The structure was refined to R_work_ = 21.68% and R_free_ = 25.68%. The crystallographic and refinement statistics are summarized in Table 1.

**Table 1:** Data collection and refinement statistics.

Data collection	
Space group	C 1 2 1
Unit cell parameter *a*, *b*, *c* (Å)	
*a*, *b*, *c* (Å)	*a* = 191.49, *b* = 55.61, *c* = 90.95
*α*, *β*, *γ* (°)	*α* = 90, *β* = 107.67, *γ* = 90
Resolution range (Å)^a^	29.44–3.29 (3.4–3.29)
Total reflections	82,495
Unique reflections	13,878 (678)
Multiplicity	5.9 (5.3)
Completeness (%)^a^	98.9 (95.1)
Mean *I*/σ(*I*)^a^	5.72 (2.15)
*R*_merge_ (%)^a^^,^ ^b^	23.2 (54.3)
**Refinement**	
Resolution range (Å)	29.45–3.29
*R*_work_ (%)	21.68
*R*_free_ (%)	25.68
No. of molecules in the asymmetric unit	2
No. of non-hydrogen atoms	7,037
Macromolecules	7,037
Solvent	0
Average *B*-factor values (Å^2^)	56.22
Ramachandran plot: favored / allowed/ outliers (%)	98.65 / 1.35 / 0
Rotamer outliers (%)	0
Clashscore	3.25
RMSD bonds (Å) / angles (°)	0.004 / 0.807

^a^Values for the outermost resolution shell are indicated in parentheses

^b^R_merge_ = Σ_h_ Σ_i_ |*I*(*h*)_i_ − <*I*(*h*)>|/ Σ_*h*_ Σ_*i*_ I(*h*)_*i*_, where *I*(*h*) is the observed intensity of reflection h and <*I*(*h*)> is the average intensity obtained from multiple measurements.

**Fig 2:**
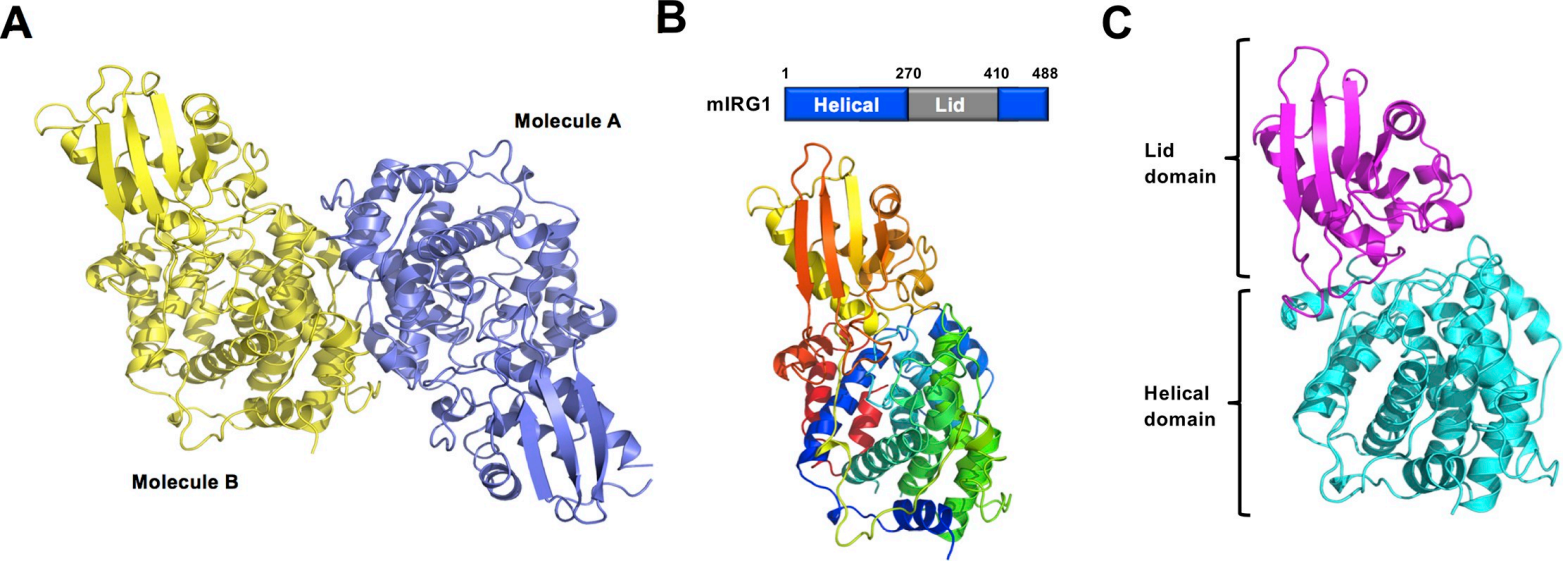
Crystal structure of mouse IRG1. (A) Cartoon representation of dimeric mouse IRG1. (B) Domain boundary of mouse IRG1. The positions of the helical domain and the lid domain are indicated using a bar diagram. A rainbow-colored cartoon representation of monomeric mouse IRG1 is shown in the lower panel. The chain from the N- to C-terminus is colored blue to red. (C) Cartoon representation showing the two distinct domains of mouse IRG1.

The detected asymmetric unit contained two molecules (A and B), which comprise the functional unit of IRG1. Following the refinement and model building process, the model of molecule A was constructed from residues 5 to 463 and the model of molecule B was constructed from residues 4 to 460 (Fig 2A). Although we produced the full-length mouse IRG1 containing all 488 amino acids, ~4 N-terminal and ~20 C-terminal residues were not traceable owing to poor electron density. The mouse IRG1 structure revealed that it is composed of two distinct domains, a helical domain and lid domain (Fig 2B and 2C). The helical domain is formed by 270 N-terminal and 80 C-terminal residues, while the lid domain is formed by 140 residues in the middle of mouse IRG1 (Fig 2B).

### Structural comparison with a recently reported mouse IRG1 (PDB ID: 6R6T) structure

**Fig 3:**
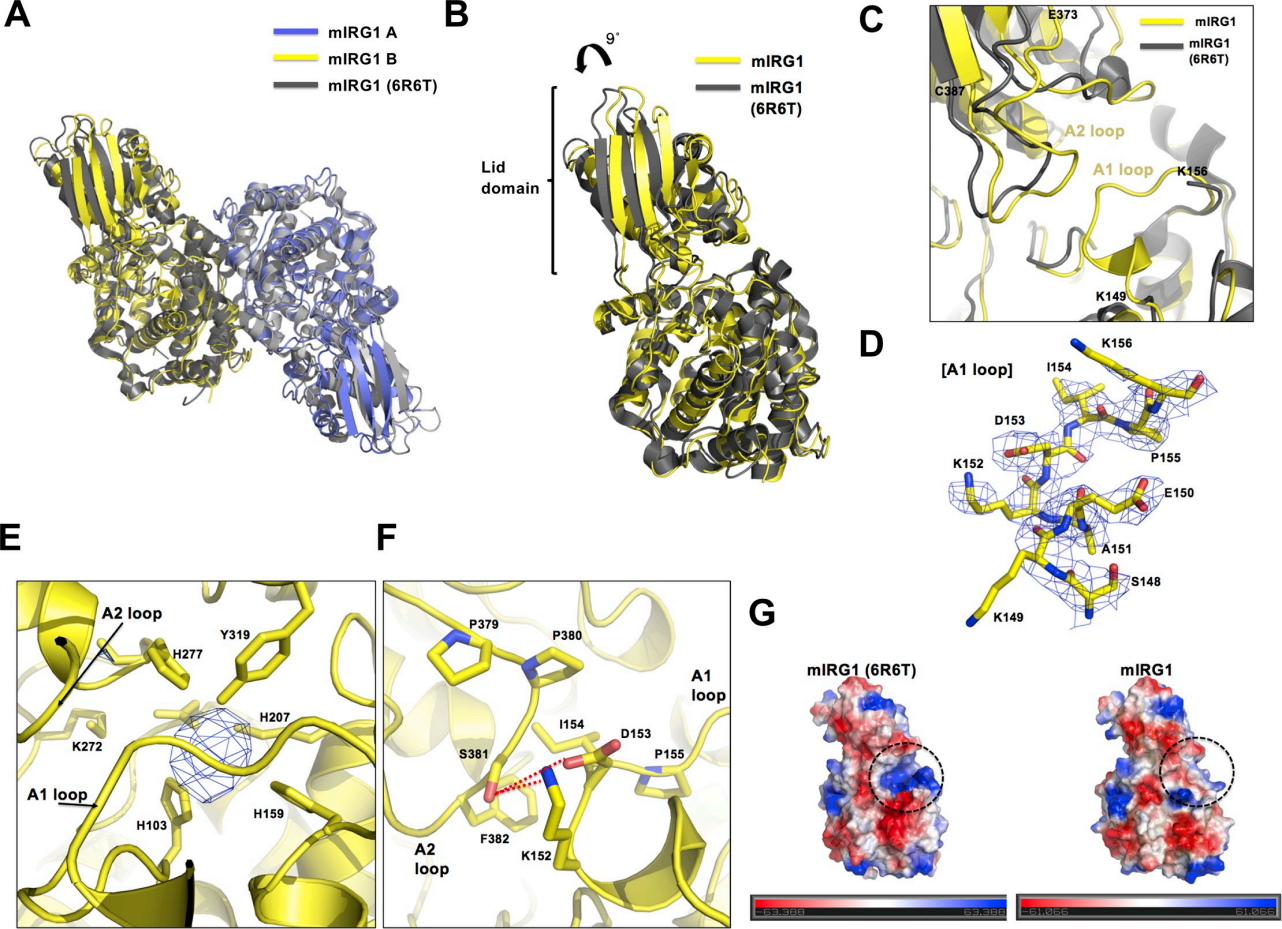
Structural comparison of mouse IRG1 with the recently reported mouse IRG1 (PDB ID: 6R6T). (A) Comparison of the dimeric structure of our mouse IRG1 with the 6R6T model by superposition analysis. (B) Superposition of the monomeric structure of our mouse IRG1 and the 6R6T model. The tilt angle of the lid domain of our mouse IRG1 is indicated above the cartoon. (C) Details of the active site of our mouse IRG1 compared with that of the 6R6T model. The two loops around the active site are labeled as A1 loop and A2 loop. (D) The 2Fo-Fc electron density map contoured at the 1.0 σ level around A1 loop. (E) Close-up view of the active site. The unknown Fo-Fc electron density contoured at the 3σ level is indicated with a blue mesh, the residues that form the active site are labeled, and the positions of the A1 and A2 loops are indicated by black arrows. (F) The interactions between A1 loop and A2 loop. Red-dash lines indicate hydrogen bonds. (G) Electrostatic surface representation of our mouse IRG1 and mouse IRG1 (6R6T). Dotted black circles indicate the active sites.

As the reported mouse IRG1 (6R6T) contains a different tag and was expressed using a different protein expression system, we compared the structure of our IRG1 with that of the recently reported mouse IRG1 (6R6T). Although the overall dimeric structures of our IRG1 and that of the 6R6T model were nearly identical, possessing an root-mean-square deviation (RMSD) of 1.9 Å (Fig 3A), the lid domain of our mouse IRG1 was tilted approximately 9° (Fig 3B), indicating that the two mouse IRG1 structures are not identical.

Structural analysis comparison indicated that the A1 loop, which is located near the active site, was not constructed in the 6R6T model because of poor electron density; in contrast, the A1 loop of our model was clearly constructed with distinct density (Fig 3C and 3D). The missing A1 loop region residues in the 6R6T model include the EAKDIP sequence from residues 147 to 155. The A2 loop structures of both models, which are composed of residues E373 to C387, were similar, although the location of the loop was slightly mismatched owing to the tilting of the lid domain (Fig 3C). Interestingly, an unknown electron density was observed in the active site of our IRG1 structure. The Fo-Fc density map contoured at a 3σ level clearly showed that this density was not due to water (Fig 3E). All the side chains of the amino acid residues forming the active site were orientated towards the unidentified density (Fig 3E). By comparing with the structure of human IRG1, we realized that hIRG1 also contains intact A1 loop similar with our mouse IRG1 [20]. Interestingly, hIRG1 containing intact A1 loop also contains unknown density around the active site [20], which is similar with the unknown density found in the active site of our mouse IRG1. Based on this observation, we hypothesized that unknown density (might be from substrate: this also suggested by Chen et al [20]) fixed the close conformation of IRG1 by holding the A1 loop. The fixed closed conformation of IRG1 was further stabilized by direct interaction of A1 and A2 loops. Structural analysis in this area showed that K152 and D153 from A1 loop formed hydrogen bonds with S381 from A2 loop (Fig 3F). Besides hydrogen bonds, hydrophobic interactions in between I154 from A1 loop, and P380 and F382 from A2 loop were also observed, indicating that A1 loop and A2 loop directly interact during the formation of closed conformation of IRG1 (Fig 3F).

The features of the surface surrounding the active site in our structure were also compared with that of the 6R6T structure by electrostatic surface calculation. As indicated at Fig 3G, mouse IRG1 (6R6T), which does not contain the A1 loop, showed a positively charged deep cavity forming active site, while our structure, which contains the A1 loop covering the active site, exhibited a hydrophobic, smooth surface in the active site region. These results indicate that the A1 loop causes the mouse IRG1 closed conformation by covering the active site and preventing substrate access.

## Discussion

The IRG1 protein, also known as CAD, is over-expressed during immune responses and catalyzes the production of itaconate, which functions as an immunometabolite possessing antibacterial, antiviral, and immunoregulatory activities [5–7, 11, 30].

To better understand the catalytic mechanism of IRG1, we performed a structural study of mouse IRG1. Our findings indicate that IRG1 exist as a dimer in solution. A decarboxylation activity test showed that mouse IRG1 and human IRG1 exhibit activity in vitro.

During the preparation of our structure analysis manuscript, the structures of another mouse IRG1 (PDB ID: 6R6T) and human IRG1 (PDB ID: 6R6U) were reported [20]. Structural comparison of our mouse IRG1 with the structure of the recently reported mouse IRG1 (6R6T) showed that in contrast with our model, the 6R6T model did not include the A1 loop around the active site because of poor electron density. This indicates that the A1 loop might be flexible and control substrate accessibility by functioning as a gate. This hypothesis led us to conclude that our mouse IRG1 is in the closed form. Indeed, recently, we solved the structure of another IRG1 from yeast; this structure was in an open form with the A1 loop located far from the active site [31]. In this study, we also showed that the lid domain, which can be tilted, might be flexible and adjust the active site, which is localized between the lid domain and the helical domain. By tilting the lid domain, A2 loop from lid domain directly interact to A1 loop of helical domain, stabilize the closed conformation of IRG1. Furthermore, an unknown density, coordinated by the surrounding residues in the active site, was detected in the center of active site; this might maintain the closed form of IRG1 containing the intact A1 loop. Previous structure of human IRG1 also contains undefined density around the active site, maintaining the intact A1 loop [20]. If the unknown density effect is the same as the substrate binding effect, it is possible that IRG1 changes to the closed conformation following substrate binding. The structure of the substrate/IRG1 complex will need to be elucidated in order to gain a better understanding of the open-closed alteration-based activity control of IRG1. Our study surely makes a significant contribution to the literature because the elucidation of activity controlling mechanism by structural changes will help in biotechnological advances to synthesize IRG1 for large scale itaconate production, which will be very useful for various industrial and biomedical applications.

## Supporting information

S1 File (PDF)

S1 Data (PDB)

## References

[1] Michelucci A, Cordes T, Ghelfi J, Pailot A, Reiling N, Goldmann O, et al Immune-responsive gene 1 protein links metabolism to immunity by catalyzing itaconic acid production. P Natl Acad Sci USA. 2013;110(19):7820–5. 10.1073/pnas.1218599110 WOS:000319327700068. 23610393 PMC3651434

[2] Daniels BP, Kofman SB, Smith JR, Norris GT, Snyder AG, Kolb JP, et al The Nucleotide Sensor ZBP1 and Kinase RIPK3 Induce the Enzyme IRG1 to Promote an Antiviral Metabolic State in Neurons. Immunity. 2019;50(1):64–+. 10.1016/j.immuni.2018.11.017 WOS:000455661600011. 30635240 PMC6342485

[3] Nair S, Huynh JP, Lampropoulou V, Loginicheva E, Esaulova E, Gounder AP, et al Irg1 expression in myeloid cells prevents immunopathology during M-tuberculosis infection. J Exp Med. 2018;215(4):1035–45. 10.1084/jem.20180118 WOS:000440817800005. 29511063 PMC5881474

[4] Kurian JV. A new polymer platform for the future—Sorona (R) from corn derived 1,3-propanediol. J Polym Environ. 2005;13(2):159–67. 10.1007/s10924-005-2947-7 WOS:000229626300008.

[5] Cordes T, Michelucci A, Hiller K. Itaconic Acid: The Surprising Role of an Industrial Compound as a Mammalian Antimicrobial Metabolite. Annu Rev Nutr. 2015;35:451–73. 10.1146/annurev-nutr-071714-034243 WOS:000358259600015. 25974697

[6] O'Neill LAJ, Artyomov MN. Itaconate: The poster child of metabolic reprogramming in macrophage function. Nat Rev Immunol. 2019;19(5):273–81. Epub 2019/02/02. 10.1038/s41577-019-0128-5 . 30705422

[7] Dominguez-Andres J, Novakovic B, Li Y, Scicluna BP, Gresnigt MS, Arts RJW, et al The Itaconate Pathway Is a Central Regulatory Node Linking Innate Immune Tolerance and Trained Immunity. Cell Metab. 2019;29(1):211–+. 10.1016/j.cmet.2018.09.003 WOS:000455090100022. 30293776

[8] Khan FR, Mcfadden BA. Enzyme Profiles in Seedling Development and the Effect of Itaconate, an Isocitrate Lyase-Directed Reagent. Plant Physiol. 1979;64(2):228–31. 10.1104/pp.64.2.228 WOS:A1979HH15900014. 16660938 PMC543060

[9] Sakai A, Kusumoto A, Kiso Y, Furuya E. Itaconate reduces visceral fat by inhibiting fructose 2,6-bisphosphate synthesis in rat liver. Nutrition. 2004;20(11–12):997–1002. 10.1016/j.nut.2004.08.007 WOS:000225337700008. 15561490

[10] Li A, Pfelzer N, Zuijderwijk R, Punt P. Enhanced itaconic acid production in Aspergillus niger using genetic modification and medium optimization. Bmc Biotechnol. 2012;12 Artn 57 10.1186/1472-6750-12-57 WOS:000309911600001. 22925689 PMC3472327

[11] Lampropoulou V, Sergushichev A, Bambouskova M, Nair S, Vincent EE, Loginicheva E, et al Itaconate Links Inhibition of Succinate Dehydrogenase with Macrophage Metabolic Remodeling and Regulation of Inflammation. Cell Metab. 2016;24(1):158–66. 10.1016/j.cmet.2016.06.004 WOS:000380793400020. 27374498 PMC5108454

[12] Dwiarti L, Yamane K, Yamatani H, Kahar P, Okabe M. Purification and characterization of cis-aconitic acid decarboxylase from Aspergillus terreus TN484-M1. J Biosci Bioeng. 2002;94(1):29–33. 10.1263/jbb.94.29 WOS:000178091600005. 16233265

[13] Kanamasa S, Dwiarti L, Okabe M, Park EY. Cloning and functional characterization of the cis-aconitic acid decarboxylase (CAD) gene from Aspergillus terreus. Appl Microbiol Biot. 2008;80(2):223–9. 10.1007/s00253-008-1523-1 WOS:000257911500004. 18584171

[14] Mills EL, Ryan DG, Prag HA, Dikovskaya D, Menon D, Zaslona Z, et al Itaconate is an anti-inflammatory metabolite that activates Nrf2 via alkylation of KEAP1. Nature. 2018;556(7699):113–+. 10.1038/nature25986 WOS:000429103300045. 29590092 PMC6047741

[15] Basler T, Jeckstadt S, Valentin-Weigand P, Goethe R. Mycobacterium paratuberculosis, Mycobacterium smegmatis, and lipopolysaccharide induce different transcriptional and post-transcriptional regulation of the IRG1 gene in murine macrophages. J Leukoc Biol. 2006;79(3):628–38. Epub 2006/01/18. 10.1189/jlb.0905520 . 16415166

[16] Strelko CL, Lu WY, Dufort FJ, Seyfried TN, Chiles TC, Rabinowitz JD, et al Itaconic Acid Is a Mammalian Metabolite Induced during Macrophage Activation. J Am Chem Soc. 2011;133(41):16386–9. 10.1021/ja2070889 WOS:000295997500018. 21919507 PMC3216473

[17] Pessler F, Mayer CT, Jung SM, Behrens EM, Dai L, Menetski JP, et al Identification of novel monosodium urate crystal regulated mRNAs by transcript profiling of dissected murine air pouch membranes. Arthritis Res Ther. 2008;10(3). ARTN R64 10.1186/ar2435 WOS:000259633000025. 18522745 PMC2483455

[18] Michopoulos F, Karagianni N, Whalley NM, Firth MA, Nikolaou C, Wilson ID, et al Targeted Metabolic Profiling of the Tg197 Mouse Model Reveals Itaconic Acid as a Marker of Rheumatoid Arthritis. J Proteome Res. 2016;15(12):4579–90. 10.1021/acs.jproteome.6b00654 WOS:000389396500035. 27704840

[19] Weiss JM, Davies LC, Karwan M, Ileva L, Ozaki MK, Cheng RY, et al Itaconic acid mediates crosstalk between macrophage metabolism and peritoneal tumors. J Clin Invest. 2018;128(9):3794–805. Epub 2018/06/20. 10.1172/JCI99169 29920191 PMC6118601

[20] Chen FF, Lukat P, Iqbal AA, Saile K, Kaever V, van den Heuvel J, et al Crystal structure of cis-aconitate decarboxylase reveals the impact of naturally occurring human mutations on itaconate synthesis. P Natl Acad Sci USA. 2019;116(41):20644–54. 10.1073/pnas.1908770116 WOS:000489770700059. 31548418 PMC6789909

[21] Vuoristo KS, Mars AE, van Loon S, Orsi E, Eggink G, Sanders JP, et al Heterologous expression of Mus musculus immunoresponsive gene 1 (irg1) in Escherichia coli results in itaconate production. Front Microbiol. 2015;6:849 Epub 2015/09/09. 10.3389/fmicb.2015.00849 26347730 PMC4539527

[22] Huang XN, Lu XF, Li YM, Li X, Li JJ. Improving itaconic acid production through genetic engineering of an industrial Aspergillus terreus strain. Microb Cell Fact. 2014;13 ARTN 119 10.1186/s12934-014-0119-y WOS:000340803600001. 25162789 PMC4251695

[23] Otwinowski Z, Minor W. Processing of X-ray diffraction data collected in oscillation mode. Methods Enzymol. 1997;276:307–26. Epub 1997/01/01. . 27754618 10.1016/S0076-6879(97)76066-X

[24] McCoy AJ. Solving structures of protein complexes by molecular replacement with Phaser. Acta Crystallogr D Biol Crystallogr. 2007;63(Pt 1):32–41. 10.1107/S0907444906045975 17164524 PMC2483468

[25] Lohkamp B, Bauerle B, Rieger PG, Schneider G. Three-dimensional structure of iminodisuccinate epimerase defines the fold of the MmgE/PrpD protein family. J Mol Biol. 2006;362(3):555–66. Epub 2006/08/29. 10.1016/j.jmb.2006.07.051 . 16934291

[26] Adams PD, Afonine PV, Bunkoczi G, Chen VB, Davis IW, Echols N, et al PHENIX: a comprehensive Python-based system for macromolecular structure solution. Acta Crystallogr D Biol Crystallogr. 2010;66(Pt 2):213–21. 10.1107/S0907444909052925 20124702 PMC2815670

[27] Emsley P, Cowtan K. Coot: model-building tools for molecular graphics. Acta Crystallogr D Biol Crystallogr. 2004;60(Pt 12 Pt 1):2126–32. 10.1107/S0907444904019158 . 15572765

[28] Chen VB, Arendall WB 3rd, Headd JJ, Keedy DA, Immormino RM, Kapral GJ, et al MolProbity: all-atom structure validation for macromolecular crystallography. Acta Crystallogr D Biol Crystallogr. 2010;66(Pt 1):12–21. Epub 2010/01/09. 10.1107/S0907444909042073 20057044 PMC2803126

[29] DeLano WL, Lam JW. PyMOL: A communications tool for computational models. Abstr Pap Am Chem S. 2005;230:U1371–U2. WOS:000236797302763.

[30] Ryan DG, Murphy MP, Frezza C, Prag HA, Chouchani ET, O'Neill LA, et al Coupling Krebs cycle metabolites to signalling in immunity and cancer. Nat Metab. 2019;1(1):16–33. 10.1038/s42255-018-0014-7 WOS:000500725600008. 31032474 PMC6485344

[31] Chun HL, Lee SY, Lee SH, Lee CS, Park HH. Enzymatic reaction mechanism of cis-aconitate decarboxylase based on the crystal structure of IRG1 from Bacillus subtilis. Sci Rep. 2020;10(1):11305 Epub 2020/07/11. 10.1038/s41598-020-68419-y 32647315 PMC7347537

