# Supplementary material for: The crystal structure of mouse IRG1 suggests that cis-aconitate decarboxylase has an open and closed conformation
Source: PLoS One. 2020 Dec 1;15(12):e0242383. doi: 10.1371/journal.pone.0242383 (PMC7707506; doi:10.1371/journal.pone.0242383)
Supplement: S1 File — (PDF) [file pone.0242383.s001.pdf]

Preliminary Full wwPDB X-ray Structure Validation Report (i)

PRELIMINARY VALIDATION REPORT

Oct 5, 2020 – 01:31 PM JST

Deposition ID : D_1300016223

This is a Preliminary Full wwPDB X-ray Structure Validation Report.

This report is produced by the wwPDB Deposition System during initial deposition but before annotation of the structure.

We welcome your comments at *validation@mail.wwpdb.org*
A user guide is available at
https://www.wwpdb.org/validation/2017/XrayValidationReportHelp
with specific help available everywhere you see the (i) symbol.

The following versions of software and data (see references (i)) were used in the production of this report:

MolProbity	:	4.02b-467
Xtriage (Phenix)	:	1.13
EDS	:	2.14.6
Percentile statistics	:	20191225.v01 (using entries in the PDB archive December 25th 2019)
Refmac	:	5.8.0158
CCP4	:	7.0.044 (Gargrove)
Ideal geometry (proteins)	:	Engh & Huber (2001)
Ideal geometry (DNA, RNA)	:	Parkinson et al. (1996)
Validation Pipeline (wwPDB-VP)	:	2.14.6

1 Overall quality at a glance (i)

The following experimental techniques were used to determine the structure:
X-RAY DIFFRACTION

The reported resolution of this entry is 3.29 Å.

Percentile scores (ranging between 0-100) for global validation metrics of the entry are shown in the following graphic. The table shows the number of entries on which the scores are based.

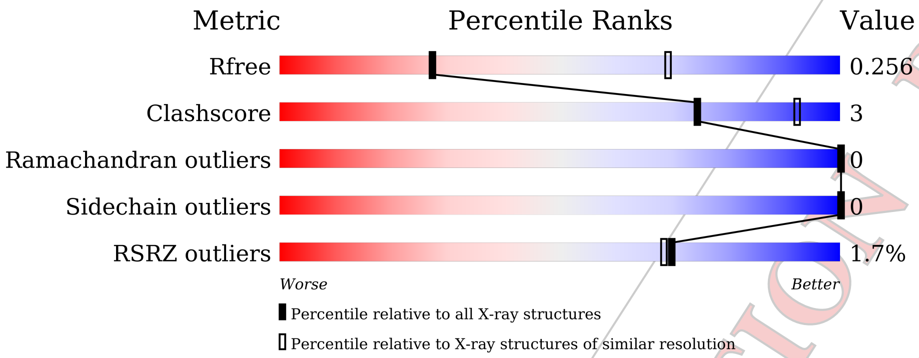

Metric	Whole archive (#Entries)	Similar resolution (#Entries, resolution range(Å))
R_{free}	130704	1149 (3.34-3.26)
Clashscore	141614	1205 (3.34-3.26)
Ramachandran outliers	138981	1183 (3.34-3.26)
Sidechain outliers	138945	1182 (3.34-3.26)
RSRZ outliers	127900	1115 (3.34-3.26)

The table below summarises the geometric issues observed across the polymeric chains and their fit to the electron density. The red, orange, yellow and green segments on the lower bar indicate the fraction of residues that contain outliers for >=3, 2, 1 and 0 types of geometric quality criteria respectively. A grey segment represents the fraction of residues that are not modelled. The numeric value for each fraction is indicated below the corresponding segment, with a dot representing fractions <=5% The upper red bar (where present) indicates the fraction of residues that have poor fit to the electron density. The numeric value is given above the bar.

Mol	Chain	Length	Quality of chain
1	A	447	2% 93% 7%
2	B	453	% 90% 10%

PRELIMINARY VALIDATION REPORT

2 Entry composition (i)

There are 2 unique types of molecules in this entry. The entry contains 6986 atoms, of which 0 are hydrogens and 0 are deuteriums.

In the tables below, the ZeroOcc column contains the number of atoms modelled with zero occupancy, the AltConf column contains the number of residues with at least one atom in alternate conformation and the Trace column contains the number of residues modelled with at most 2 atoms.

- Molecule 1 is a protein called Cis-aconitate decarboxylase.

Mol	Chain	Residues	Atoms					ZeroOcc	AltConf	Trace
			Total	C	N	O	S			
1	A	447	3466	2209	607	638	12	0	0	0

- Molecule 2 is a protein called Cis-aconitate decarboxylase.

Mol	Chain	Residues	Atoms					ZeroOcc	AltConf	Trace
			Total	C	N	O	S			
2	B	453	3520	2246	615	647	12	0	0	0

PRELIMINARY VALIDATION REPORT

3 Residue-property plots (i)

These plots are drawn for all protein, RNA, DNA and oligosaccharide chains in the entry. The first graphic for a chain summarises the proportions of the various outlier classes displayed in the second graphic. The second graphic shows the sequence view annotated by issues in geometry and electron density. Residues are color-coded according to the number of geometric quality criteria for which they contain at least one outlier: green = 0, yellow = 1, orange = 2 and red = 3 or more. A red dot above a residue indicates a poor fit to the electron density (RSRZ > 2). Stretches of 2 or more consecutive residues without any outlier are shown as a green connector. Residues present in the sample, but not in the model, are shown in grey.

- Molecule 1: Cis-aconitate decarboxylase

Chain A:

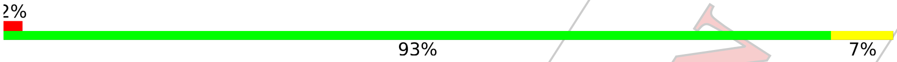

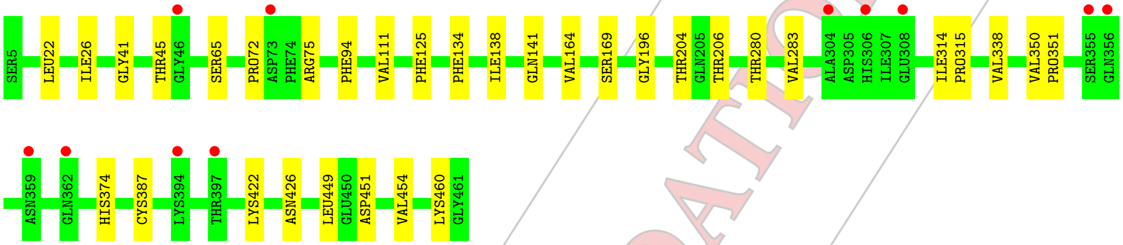

- Molecule 2: Cis-aconitate decarboxylase

Chain B:

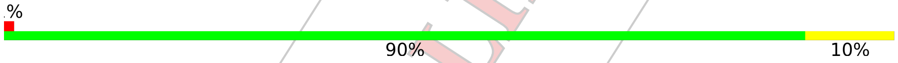

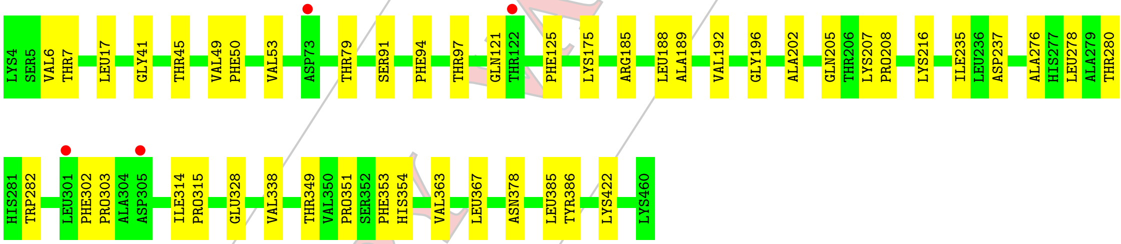

PRELIMINARY VALIDATION REPORT

4 Data and refinement statistics (i)

Property	Value	Source
Space group	C 1 2 1	Depositor
Cell constants<br>a, b, c, α, β, γ	191.49Å 55.61Å 90.96Å<br>90.00° 107.67° 90.00°	Depositor
Resolution (Å)	29.45 – 3.29<br>29.45 – 3.29	Depositor<br>EDS
% Data completeness<br>(in resolution range)	98.1 (29.45-3.29)<br>98.1 (29.45-3.29)	Depositor<br>EDS
R_{merge}	(Not available)	Depositor
R_{sym}	0.16	Depositor
$< I/\sigma(I) >$ [1]	2.49 (at 3.31Å)	Xtriage
Refinement program	PHENIX (1.18.2_3874: ???)	Depositor
R, R_{free}	0.217 , 0.257<br>0.216 , 0.256	Depositor<br>DCC
R_{free} test set	1385 reflections (9.98%)	wwPDB-VP
Wilson B-factor (Å^2)	33.3	Xtriage
Anisotropy	0.673	Xtriage
Bulk solvent k_{sol}(e/Å^3), B_{sol}(Å^2)	0.36 , 32.0	EDS
L-test for twinning[2]	$< \|L\| > = 0.48$, $< L^2 > = 0.30$	Xtriage
Estimated twinning fraction	No twinning to report.	Xtriage
F_o,F_c correlation	0.87	EDS
Total number of atoms	6986	wwPDB-VP
Average B, all atoms (Å^2)	21.0	wwPDB-VP

Xtriage's analysis on translational NCS is as follows: *The largest off-origin peak in the Patterson function is 4.78% of the height of the origin peak. No significant pseudotranslation is detected.*

[1] Intensities estimated from amplitudes.

[2] Theoretical values of $< |L| >$, $< L^2 >$ for acentric reflections are 0.5, 0.333 respectively for untwinned datasets, and 0.375, 0.2 for perfectly twinned datasets.

PRELIMINARY VALIDATION REPORT

5 Model quality (i)

5.1 Standard geometry (i)

The Z score for a bond length (or angle) is the number of standard deviations the observed value is removed from the expected value. A bond length (or angle) with $|Z| > 5$ is considered an outlier worth inspection. RMSZ is the root-mean-square of all Z scores of the bond lengths (or angles).

Mol	Chain	Bond lengths		Bond angles	
		RMSZ	#$|Z|$ >5	RMSZ	#$|Z|$ >5
1	A	0.28	0/3552	0.50	0/4823
2	B	0.28	0/3609	0.51	0/4904
All	All	0.28	0/7161	0.51	0/9727

There are no bond length outliers.

There are no bond angle outliers.

There are no chirality outliers.

There are no planarity outliers.

5.2 Too-close contacts (i)

In the following table, the Non-H and H(model) columns list the number of non-hydrogen atoms and hydrogen atoms in the chain respectively. The H(added) column lists the number of hydrogen atoms added and optimized by MolProbity. The Clashes column lists the number of clashes within the asymmetric unit, whereas Symm-Clashes lists symmetry related clashes.

Mol	Chain	Non-H	H(model)	H(added)	Clashes	Symm-Clashes
1	A	3466	0	3448	18	0
2	B	3520	0	3507	27	0
All	All	6986	0	6955	44	0

The all-atom clashscore is defined as the number of clashes found per 1000 atoms (including hydrogen atoms). The all-atom clashscore for this structure is 3.

All (44) close contacts within the same asymmetric unit are listed below, sorted by their clash magnitude.

Atom-1	Atom-2	Interatomic distance (Å)	Clash overlap (Å)
2:B:378:ASN:HB3	2:B:386:TYR:CE2	2.26	0.71
1:A:196:GLY:HA3	2:B:196:GLY:HA3	1.74	0.70

Continued on next page...

PRELIMINARY VALIDATION REPORT

Continued from previous page...

Atom-1	Atom-2	Interatomic distance (Å)	Clash overlap (Å)
1:A:72:PRO:HB3	1:A:460:LYS:O	1.95	0.67
1:A:94:PHE:CZ	1:A:422:LYS:HE3	2.31	0.66
1:A:314:ILE:HG13	1:A:315:PRO:HD2	1.83	0.60
1:A:204:THR:HG23	1:A:206:THR:H	1.68	0.56
2:B:189:ALA:HA	2:B:235:ILE:HG21	1.89	0.55
2:B:349:THR:HG23	2:B:351:PRO:HD2	1.88	0.54
2:B:314:ILE:HG13	2:B:315:PRO:HD2	1.90	0.54
1:A:94:PHE:HE2	1:A:426:ASN:HD21	1.56	0.54
2:B:121:GLN:HG2	2:B:125:PHE:CZ	2.45	0.52
1:A:280:THR:HB	1:A:338:VAL:HG21	1.92	0.51
1:A:449:LEU:HD11	1:A:454:VAL:HB	1.94	0.50
2:B:6:VAL:HB	2:B:237:ASP:HB3	1.95	0.49
2:B:7:THR:HG22	2:B:185:ARG:HG3	1.96	0.47
1:A:41:GLY:O	1:A:45:THR:HG23	2.15	0.47
1:A:283:VAL:HA	1:A:387:CYS:SG	2.55	0.47
2:B:202:ALA:O	2:B:207:LYS:HD2	2.16	0.46
2:B:280:THR:HB	2:B:338:VAL:HG21	1.98	0.46
2:B:302:PHE:CD1	2:B:303:PRO:HD2	2.52	0.45
2:B:328:GLU:HG2	2:B:353:PHE:O	2.17	0.45
1:A:125:PHE:O	1:A:451:ASP:HA	2.17	0.45
2:B:50:PHE:HA	2:B:53:VAL:HG22	1.98	0.45
2:B:188:LEU:O	2:B:192:VAL:HG23	2.18	0.44
2:B:41:GLY:O	2:B:45:THR:HG23	2.18	0.44
2:B:17:LEU:O	2:B:175:LYS:NZ	2.37	0.44
2:B:276:ALA:HB2	2:B:353:PHE:CZ	2.52	0.44
2:B:282:TRP:CD2	2:B:385:LEU:HD22	2.53	0.43
2:B:49:VAL:HG22	2:B:205:GLN:HG3	2.01	0.42
2:B:363:VAL:O	2:B:367:LEU:HB2	2.19	0.42
2:B:79:THR:CG2	2:B:216:LYS:HG3	2.50	0.42
2:B:94:PHE:CZ	2:B:422:LYS:HE3	2.55	0.42
1:A:111:VAL:HG21	1:A:169:SER:OG	2.20	0.42
2:B:91:SER:HA	2:B:208:PRO:CG	2.49	0.42
1:A:65:SER:HB2	1:A:75:ARG:HB3	2.02	0.41
1:A:22:LEU:HA	1:A:26:ILE:HD11	2.02	0.41
1:A:141:GLN:HG2	1:A:164:VAL:O	2.20	0.41
2:B:49:VAL:HG22	2:B:205:GLN:CG	2.50	0.41
1:A:134:PHE:O	1:A:138:ILE:HG12	2.20	0.41
1:A:350:VAL:HB	1:A:351:PRO:HD3	2.02	0.41
1:A:314:ILE:O	1:A:374:HIS:ND1	2.45	0.41
2:B:97:THR:HG21	2:B:278:LEU:HD22	2.03	0.41
2:B:237:ASP:N	2:B:237:ASP:OD1	2.54	0.41

Continued on next page...

PRELIMINARY VALIDATION REPORT

Continued from previous page...

Atom-1	Atom-2	Interatomic distance (Å)	Clash overlap (Å)
2:B:351:PRO:HA	2:B:354:HIS:CE1	2.56	0.41

There are no symmetry-related clashes.

5.3 Torsion angles (i)

5.3.1 Protein backbone (i)

In the following table, the Percentiles column shows the percent Ramachandran outliers of the chain as a percentile score with respect to all X-ray entries followed by that with respect to entries of similar resolution.

The Analysed column shows the number of residues for which the backbone conformation was analysed, and the total number of residues.

Mol	Chain	Analysed	Favoured	Allowed	Outliers	Percentiles	
1	A	441/447 (99%)	436 (99%)	5 (1%)	0	100	100
2	B	449/453 (99%)	442 (98%)	7 (2%)	0	100	100
All	All	890/900 (99%)	878 (99%)	12 (1%)	0	100	100

There are no Ramachandran outliers to report.

5.3.2 Protein sidechains (i)

In the following table, the Percentiles column shows the percent sidechain outliers of the chain as a percentile score with respect to all X-ray entries followed by that with respect to entries of similar resolution.

The Analysed column shows the number of residues for which the sidechain conformation was analysed, and the total number of residues.

Mol	Chain	Analysed	Rotameric	Outliers	Percentiles	
1	A	377/377 (100%)	377 (100%)	0	100	100
2	B	384/384 (100%)	384 (100%)	0	100	100
All	All	761/761 (100%)	761 (100%)	0	100	100

There are no protein residues with a non-rotameric sidechain to report.

Some sidechains can be flipped to improve hydrogen bonding and reduce clashes. There are no such sidechains identified.

PRELIMINARY VALIDATION REPORT

5.3.3 RNA (i)

There are no RNA molecules in this entry.

5.4 Non-standard residues in protein, DNA, RNA chains (i)

There are no non-standard protein/DNA/RNA residues in this entry.

5.5 Carbohydrates (i)

There are no monosaccharides in this entry.

5.6 Ligand geometry (i)

There are no ligands in this entry.

5.7 Other polymers (i)

There are no such residues in this entry.

5.8 Polymer linkage issues (i)

The following chains have linkage breaks:

Mol	Chain	Number of breaks
1	A	2
2	B	1

All chain breaks are listed below:

Model	Chain	Residue-1	Atom-1	Residue-2	Atom-2	Distance (Å)
1	A	295:VAL	C	303:PRO	N	7.84
1	B	296:THR	C	301:LEU	N	6.60
1	A	120:PRO	C	124:LYS	N	5.97

PRELIMINARY VALIDATION REPORT

6 Fit of model and data (i)

6.1 Protein, DNA and RNA chains (i)

In the following table, the column labelled '#RSRZ> 2' contains the number (and percentage) of RSRZ outliers, followed by percent RSRZ outliers for the chain as percentile scores relative to all X-ray entries and entries of similar resolution. The OWAB column contains the minimum, median, 95th percentile and maximum values of the occupancy-weighted average B-factor per residue. The column labelled 'Q< 0.9' lists the number of (and percentage) of residues with an average occupancy less than 0.9.

Mol	Chain	Analysed	<RSRZ>	#RSRZ>2			OWAB(Å^2)	Q<0.9
1	A	447/447 (100%)	0.00	11 (2%)	57	54	9, 21, 50, 81	0
2	B	453/453 (100%)	-0.20	4 (0%)	84	84	9, 16, 37, 66	0
All	All	900/900 (100%)	-0.10	15 (1%)	70	68	9, 18, 46, 81	0

All (15) RSRZ outliers are listed below:

Mol	Chain	Res	Type	RSRZ
2	B	122	THR	3.9
2	B	305	ASP	3.8
1	A	397	THR	3.2
1	A	306	HIS	3.0
1	A	362	GLN	2.9
1	A	304	ALA	2.7
2	B	301	LEU	2.6
1	A	356	GLN	2.5
1	A	394	LYS	2.5
1	A	46	GLY	2.5
1	A	355	SER	2.2
1	A	73	ASP	2.2
1	A	359	ASN	2.1
2	B	73	ASP	2.1
1	A	308	GLU	2.0

6.2 Non-standard residues in protein, DNA, RNA chains (i)

There are no non-standard protein/DNA/RNA residues in this entry.

6.3 Carbohydrates (i)

There are no monosaccharides in this entry.

PRELIMINARY VALIDATION REPORT

6.4 Ligands (i)

There are no ligands in this entry.

6.5 Other polymers (i)

There are no such residues in this entry.

PRELIMINARY VALIDATION REPORT
